# Amplicon-Based Detection and Sequencing of SARS-CoV-2 in Nasopharyngeal Swabs from Patients With COVID-19 and Identification of Deletions in the Viral Genome That Encode Proteins Involved in Interferon Antagonism

**DOI:** 10.3390/v12101164

**Published:** 2020-10-14

**Authors:** Shona C. Moore, Rebekah Penrice-Randal, Muhannad Alruwaili, Nadine Randle, Stuart Armstrong, Catherine Hartley, Sam Haldenby, Xiaofeng Dong, Abdulrahman Alrezaihi, Mai Almsaud, Eleanor Bentley, Jordan Clark, Isabel García-Dorival, Paul Gilmore, Ximeng Han, Benjamin Jones, Lisa Luu, Parul Sharma, Ghada Shawli, Yani Sun, Qin Zhao, Steven T. Pullan, Daniel P. Carter, Kevin Bewley, Jake Dunning, En-min Zhou, Tom Solomon, Michael Beadsworth, James Cruise, Derrick W. Crook, David A. Matthews, Andrew D. Davidson, Zana Mahmood, Waleed Aljabr, Julian Druce, Richard Vipond, Lisa Ng, Laurent Renia, Peter J. M. Openshaw, J. Kenneth Baillie, Miles W. Carroll, James Stewart, Alistair Darby, Malcolm Semple, Lance Turtle, Julian A. Hiscox

**Affiliations:** 1 Institute of Infection, Veterinary and Ecological Sciences, University of Liverpool, Liverpool L3 5RF, UK; Shona.Moore@liverpool.ac.uk (S.C.M.); hlrpenri@liverpool.ac.uk (R.P.-R.); Muhannad.Alruwaili@liverpool.ac.uk (M.A); nprandle@liverpool.ac.uk (N.R.); sarmstro@liverpool.ac.uk (S.A.); csguy@liverpool.ac.uk (C.H.); haldenby@liverpool.ac.uk (S.H.); Xiaofeng.Dong2@liverpool.ac.uk (X.D.); A.Alrezaihi@liverpool.ac.uk (A.A.); Mai.Almsaud@liverpool.ac.uk (M.A.); hlebentl@student.liverpool.ac.uk (E.B.); Jordan.Clark@liverpool.ac.uk (J.C.); isagardo@liverpool.ac.uk (I.G.-D.); gilmore@liverpool.ac.uk (P.G.); Ximeng.Han@liverpool.ac.uk (X.H.); Benjamin.Jones3@liverpool.ac.uk (B.J.); lisaluu@liverpool.ac.uk (L.L.); parul13@liverpool.ac.uk (P.S.); G.Shawli@liverpool.ac.uk (G.S.); Yani.Sun@liverpool.ac.uk (Y.S.); Qin.Zhao@liverpool.ac.uk (Q.Z.); tsolomon@liverpool.ac.uk (T.S.); Z.Mahmood2@liverpool.ac.uk (Z.M.); waljabr@kfmc.med.sa (W.A.); lisa_ng@immunol.a-star.edu.sg (L.N.); jpstewar@liverpool.ac.uk (J.S.); acdarby@liverpool.ac.uk (A.D.); castle@liverpool.ac.uk (M.S.); 2 College of Veterinary Medicine, Northwest A&F University, Yangling 712100, China; zhouem@nwsuaf.edu.cn; 3 National Infection Service, Public Health England, Salisbury SP4 0JG, UK; Steven.Pullan@phe.gov.uk (S.T.P.); daniel.carter@phe.gov.uk (D.P.C.); kevin.bewley@phe.gov.uk (K.B.); jake.dunning@phe.gov.uk (J.D.); richard.vipond@phe.gov.uk (R.V.); Miles.Carroll@phe.gov.uk (M.W.C.); 4 Health Protection Research Unit in Emerging and Zoonotic Infections, Liverpool L69 7BE, UK; 5 Liverpool Health Partners, Liverpool L3 5TF, UK; 6 Tropical & Infectious Disease Unit, Liverpool University Hospitals NHS Foundation Trust (a member of Liverpool Health Partners), Liverpool L7 8XP, UK; mike.beadsworth@rlbuht.nhs.uk (M.B.); james.cruise@liverpoolft.nhs.uk (J.C.); 7 Nuffield Department of Medicine, University of Oxford, Oxford OX3 7BN, UK; derrick.crook@ndcls.ox.ac.uk; 8 School of Cellular and Molecular Medicine, University of Bristol, Bristol BS8 1TD, UK; d.a.matthews@bristol.ac.uk (D.A.M.); Andrew.Davidson@bristol.ac.uk (A.D.D.); 9 Laboratory Department, Directorate of Veterinary in Sulaimany, Rizgari 402, Sulaimani, Kurdistan Region 46001, Iraq; 10 Research Center, King Fahad Medical City, Riyadh 11525, Saudi Arabia; 11 The Peter Doherty Institute for Infection and Immunity, Melbourne 3000, Australia; Julian.Druce@mh.org.au; 12 Infectious Diseases Horizontal Technology Centre (ID HTC), A*STAR, Singapore 138648, Singapore; renia_laurent@immunol.a-star.edu.sg; 13 Faculty of Medicine, Imperial College London, London SW7 2AZ, UK; p.openshaw@imperial.ac.uk; 14 Roslin Institute, University of Edinburgh, Edinburgh EH25 9RG, UK; j.k.baillie@ed.ac.uk

**Keywords:** SARS-CoV-2, next-generation sequencing, amplicon, MinION

## Abstract

Severe acute respiratory syndrome coronavirus 2 (SARS-CoV-2) is the causative agent of coronavirus disease 2019 (COVID-19). Sequencing the viral genome as the outbreak progresses is important, particularly in the identification of emerging isolates with different pathogenic potential and to identify whether nucleotide changes in the genome will impair clinical diagnostic tools such as real-time PCR assays. Although single nucleotide polymorphisms and point mutations occur during the replication of coronaviruses, one of the biggest drivers in genetic change is recombination. This can manifest itself in insertions and/or deletions in the viral genome. Therefore, sequencing strategies that underpin molecular epidemiology and inform virus biology in patients should take these factors into account. A long amplicon/read length-based RT-PCR sequencing approach focused on the Oxford Nanopore MinION/GridION platforms was developed to identify and sequence the SARS-CoV-2 genome in samples from patients with or suspected of COVID-19. The protocol, termed Rapid Sequencing Long Amplicons (RSLAs) used random primers to generate cDNA from RNA purified from a sample from a patient, followed by single or multiplex PCRs to generate longer amplicons of the viral genome. The base protocol was used to identify SARS-CoV-2 in a variety of clinical samples and proved sensitive in identifying viral RNA in samples from patients that had been declared negative using other nucleic acid-based assays (false negative). Sequencing the amplicons revealed that a number of patients had a proportion of viral genomes with deletions.

## 1. Introduction

Severe acute respiratory syndrome coronavirus 2 (SARS-CoV-2) emerged in China in 2019 and has sequence similarity to SARS-CoV and certain bat coronaviruses [1]. After an incubation period that averages 5 days but may be between two days and two weeks [2], patients with the disease, termed coronavirus disease 2019 (COVID-19), typically present with fever, myalgia, cough, sore throat, and difficulty breathing. A range of other symptoms are also possible, in particular gastrointestinal symptoms. Approximately 80% of infections are mild, but of those that require hospitalisation, mortality is around 30% [3,4]. SARS-CoV-2 is highly transmissible with a reproductive number (R_0_) of around 3 if unmanaged.

SARS-CoV-2 has a positive-sense RNA genome of approximately 30 kb, and viral RNA synthesis occurs in the cytoplasm of an infected cell. During RNA synthesis, several processes occur including replication of the genome and transcription of a nested set of subgenomic messenger RNAs, which encode for the suite of viral proteins, as well as the genome for ORF1AB. During viral RNA synthesis, errors can occur including point mutation of single nucleic acids and recombination [5]. Recombination events manifest themselves in insertions and/or deletions in the viral genome and can result in major changes in viral tropism. One of the unusual features of the SARS-CoV-2 genome sequence, compared to the most closely related coronaviruses, such as SARS-CoV, is the insertion of a furin cleavage site in the spike glycoprotein (S protein) sequence [6]. The origin of the furin cleavage site is unknown but may be either viral or cellular in origin. The presence of the furin cleavage site reduces the dependence on cellular proteases to process the S protein into two functional subunits, S1 and S2. Thus, recombination can result in the insertion of a non-coronavirus sequence. The furin cleavage site is potentially unstable [7].

SARS-CoV-2 has caused a worldwide pandemic and has severe economic and health implications for all affected countries. Given that mechanisms of both point mutation and recombination operate in coronaviruses, monitoring for these changes in the SARS-CoV-2 genome is essential not only for potential contact tracing but also for confidence in vaccine efficacy, which are generally based around the S protein. Experience from developing vaccines against the coronavirus infectious bronchitis virus (IBV), which arguably have been the most successful vaccines developed against coronaviruses so far, has suggested that differences of as little as 5% between the S1 sequences can result in poor cross-protection against different variants of IBV [8]. Differences in S1 of 2% to 3% (10 to 15 amino acids) can change serotype, suggesting that a small number of epitopes are immunodominant with respect to neutralizing antibody [8].

Anecdotal data from diagnostic laboratories (at least based in the UK) early in the outbreak suggested that nucleic acid-based diagnostics to SARS-CoV-2 and complications around the sampling processes can lead to unreliable results and false negatives. The advantage of both laboratory [9] and field-based sequencing [10] approaches in characterising viral infection was illustrated in the 2013–2016 West African Ebola virus outbreak, and allowed the origin of clusters of infection to be rapidly identified [11]. Therefore, the accurate identification and sequencing of SARS-CoV-2 in samples from patients provide multiple information from reducing false negatives, contact tracing, to assessing the suitability of diagnostic assays (especially nucleic acid-based) and investigating whether vaccines are likely to be or remain efficacious in the background of nucleotide substitution and recombination during virus replication. The International Severe Acute Respiratory and emerging Infection Consortium (ISARIC) 4C UK study provided a large and detailed collection of sequentially collected virological samples from well-characterised hospitalised patients; here we assess the utility of the Rapid Sequencing Long Amplicon (RSLA) protocol in monitoring viral RNA from early participants.

## 2. Materials and Methods

### 2.1. Ethics and Clinical Information

Patients (*n* = 24) were recruited under the International Severe Acute Respiratory and Emerging Infection Consortium (ISARIC) Clinical Characterisation Protocol (CCP) (https://isaric.net/ccp) by giving informed consent. ISARIC CCP was reviewed and approved by the national research ethics service, Oxford (13/SC/0149). Samples from clinical specimens were processed at CL3 in the Outbreak Response Laboratory at the University of Liverpool, Liverpool, UK.

### 2.2. RNA Extraction and Preparation

Nasopharyngeal swabs were collected into viral transport medium from patients with COVID-19. RNA was isolated using either a QIAamp Viral RNA Mini Kit (Qiagen, Mancheste, UK) by spin-column procedure or Trizol LS (Invitrogen, UK), according to the manufacturer’s instructions. Total RNA was purified from SARS-CoV-2 infected Vero cells following AVL inactivation using the Qiagen RNA Mini Kit. Infection of Vero cells was conducted at Public Health England, Porton Down at CL3. RNA samples were treated with Turbo DNase (Invitrogen).

### 2.3. Primer Design

Alignments generated from the NCBI reference sequence for SARS-CoV-2 NCBI (NC_045512.2) and published sequences on GISAID were used to identify conserved regions for primer design. Primers (Table 1) were chosen that sequentially amplified roughly 1000 bp with an ~200 bp overlapping region.

### 2.4. RT and PCR 

SuperScript IV (Invitrogen) was used to generate single-strand cDNA using random primer mix (NEB, Hitchin, UK). The primer sets were used to generate 30 amplicons from the cDNA. Subsequently, primers were pooled to generate six sets each containing five primer pairs. Reactions were performed using 2 × Q5-High Fidelity master mix (NEB) with 0.5 μM of each primer for the individual reactions and 0.1 μM of each primer for the pooled primers. The reaction conditions were as follows: denaturation at 98 °C for 30 sec followed by 35 to 40 cycles of 10 sec denaturation at 98 °C, 30 sec annealing at 66 °C and then 50 sec of extension at 72 °C. A final extension step was done for 2 min at 72 °C.

### 2.5. Library Preparation for MinION Sequencing

Following amplification, PCR products were purified at a 1:1 ratio with AMPure XP beads (Beckman Coulter, High Wycombe, UK). The library was prepared as per the sequencing by ligation protocol with native barcodes for multiplexing (Oxford Nanopore, Oxford, UK).

### 2.6. Informatics Analysis 

For assembly of the SARS-CoV-2 genomes, Minimap2 (v. 2.17-r941) was used to align fastq sequences to the coronavirus 2 isolate Wuhan-Hu-1 reference genome (NC_045512.2) using the -ax map-ont parameters. Samtools (v.1.10) was used to sort and index alignment files, and Picard (v.2.23.4) was used to mark duplicates. A custom script written in perl (v.5.26.2) was used to determine viral genome coverage, the viral consensus sequence and the presence of indels. Viral genome coverage was visualised in RStudio (v. 4.0.2). For the identification of deletions within the SARS-CoV-2 genome, ONT RSLA library reads were aligned to the coronavirus 2 isolate Wuhan-Hu-1 reference genome using Minimap2, as part of the Nextflow ARTIC analysis pipeline (https://github.com/connor-lab/ncov2019-artic-nf). Raw aligned BAM files were used as input to SVIM, to detect putative deletions. Resulting candidate deletions were filtered to accept any with at least 5 supporting reads and intersected with reference gene annotations to determine which genes intersected with the deletions. 

## 3. Results

### 3.1. Primer and Amplicon Design

**Figure 1:**
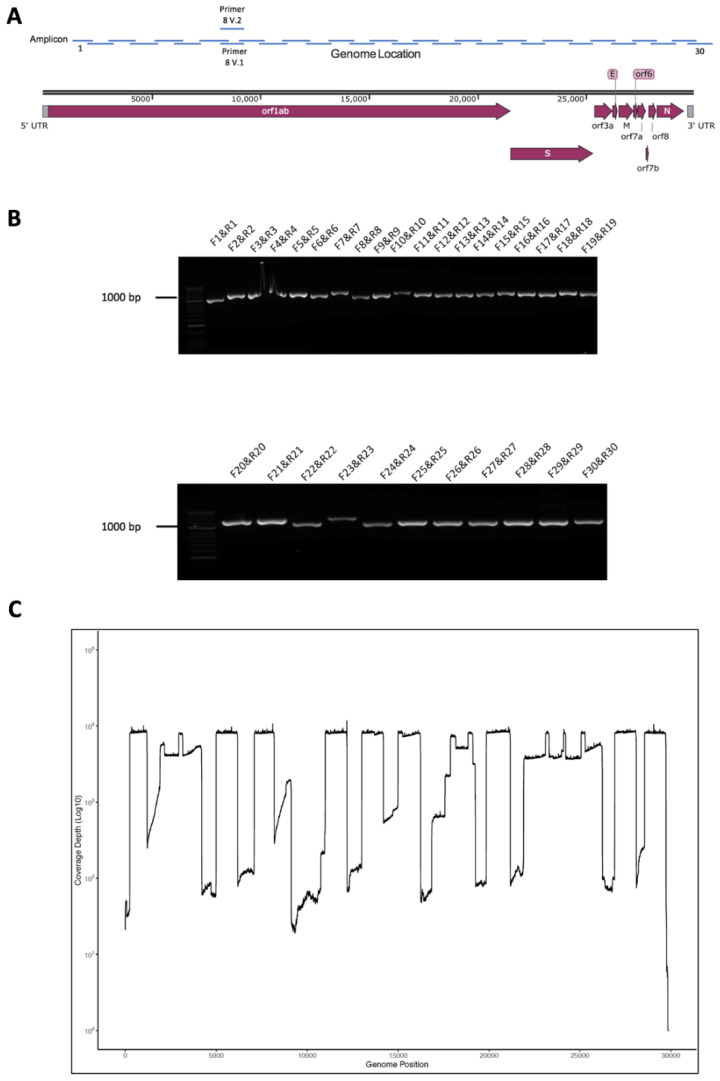
(**A**) Schematic diagram of the severe acute respiratory syndrome coronavirus 2 (SARS-CoV-2) genome showing the position of major open reading frames and the position of the amplicons along the genome. (**B**) Agarose gel electrophoresis analysis of the amplicon products resulting from RT-PCR using the designated forward and reverse primers to amplify the SARS-CoV-2 genome from RNA purified from Vero cells infected with the virus. (**C**) The amplicon products were purified and sequenced on a single flow cell using an Oxford Nanopore MinION. Shown are the number of reads that map (*y*-axis) to each amplicon across the SARS-CoV-2 genome from 5′ to 3′ (*x*-axis).

To sequence SARS-CoV-2 with a view to identify both nucleotide polymorphisms and recombination events on the viral genome, a longer read length amplicon-based system was developed. A series of primers were designed (Table 1), allowing overlapping sections of the SARS-CoV-2 genome to be amplified sequentially in 1000 base-paired fragments, with an approximately 200 base pair overlap to facilitate sequence assembly from the amplicon data (Figure 1A). The primers were selected on the basis of conserved regions in the SARS-CoV-2 genome based upon an initial deposition of 17 genomes available on GISAID.

### 3.2. Validation of Amplicon Generation and Oxford Nanopore Sequencing Using RNA from Cells Infected with SARS-CoV-2

Purified RNA from SARS-CoV-2 (MT007544.1 GenBank)-infected Vero cells was used to confirm that the primers could generate amplicons. This RNA was used as a template for cDNA synthesis followed by PCR using the conserved primers. This generated 30 separate amplicons covering the SARS-CoV-2 genome, and the synthesis and expected size of these amplicons were confirmed using agarose gel electrophoresis (Figure 1B). These amplicons were sequenced on an Oxford Nanopore (UK) flow cell using MinION and reads were mapped to the SARS-CoV-2 genome confirming that the amplicons represented viral sequence (Figure 1C). This approach was termed Rapid Sequencing Long Amplicons (RSLAs).

### 3.3. Initial Evaluation of the Amplicon Approach to Detect SARS-CoV-2 on RNA Purified from Nasopharyngeal Swabs Collected from Patients with COVID-19 Correctly Identified a Positive but also a False Negative Sample

**Figure 2:**
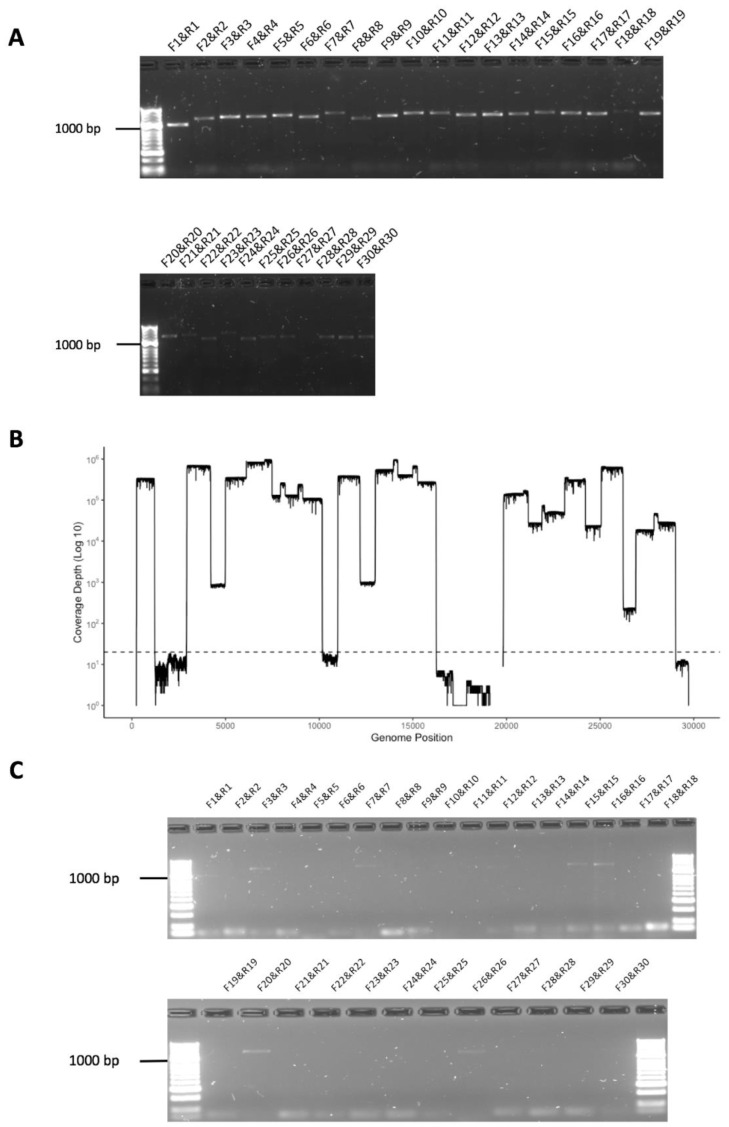
(**A**) Agarose gel electrophoresis analysis of amplicons generated by RT-PCR from RNA isolated from a nasopharyngeal swab taken from patient REMRQ0001, who had coronavirus disease 2019 (COVID-19), and diagnosed positive for SARS-CoV-2 by a laboratory-based test. Primer pairs are indicated above each amplicon. (**B**) The amplicon products were purified and sequenced on a single flow cell using an Oxford Nanopore MinION. Shown are the number of reads that map (*y*-axis) to each amplicon across the SARS-CoV-2 genome from 5′ to 3′ (*x*-axis). (**C**) Agarose gel electrophoresis analysis of amplicons generated by RT-PCR from RNA isolated from a nasopharyngeal swab taken from patient REMRQ0001, who had COVID-19, and subsequently found negative for SARS-CoV-2 by a laboratory-based test. Note that the brightness of the image has been adjusted post-image capture to more clearly show amplicon products.

The ability of the amplicon-based approach to detect SARS-CoV-2 was initially evaluated on nasopharyngeal swabs collected from patients with COVID-19. The first patient (REMRQ0001) was admitted to the Royal Liverpool University Hospital on 23 February 2020. The patient was a member of staff from the Diamond Princes cruise ship that had an outbreak of SARS-CoV-2 leading to passengers developing COVID-19 [12,13]. The patient was asymptomatic but was screened for SARS-CoV-2 on 20 February 2020, left the ship on 21 February and was repatriated from Japan on 22 February 2020. During the flight, a positive result for SARS-CoV-2 using a nucleic acid-based system was received. A repeat nasopharyngeal swab was taken on admission and again tested positive with a viral load of Ct = 31.3. Two repeat swabs were collected on 24 February, one was positive (Ct = 32.7) and the second was negative. On 25 February, a fourth diagnostic swab was again negative, but a separately collected swab yielded PCR products from RNA isolated to evaluate the amplicon system. Here, 30 separate amplicons were synthesized and then visualised by agarose gel electrophoresis (Figure 2A). The data indicated that all amplicons (F1&R1 to F30&R30) could be identified and spanned the SARS-CoV-2 genome. These amplicons were sequenced on an Oxford Nanopore flow cell using MinION and sequence reads were mapped to the SARS-CoV-2 genome (Figure 2B), confirming that the amplicons represented viral sequence.

A final nasopharyngeal swab was taken from patient REMRQ0001 on 27 February. This latter swab was not used for a diagnostic assay but was used to evaluate the sensitivity of the amplicon-based system using RNA isolated from the sample. In this case, several amplicon products were identified, though fewer than on the previous sample (note that the brightness on the gel image was adjusted post-exposure to show these products more clearly) (Figure 2C). The products most clearly visible included amplicons from the polymerase (orf1ab) region (F1&R1, F3&R3, F7&R7, F12&R12, F15&R15, F16&R16, F20&R20) and the membrane (M) region (F26&R26). Other amplicon products were less visible including F28&R28, spanning the orf8/nucleoprotein gene sequence. Therefore, the data suggested that the viral genome or viral sequence was present in a patient that had been evaluated as negative.

**Figure 3:**
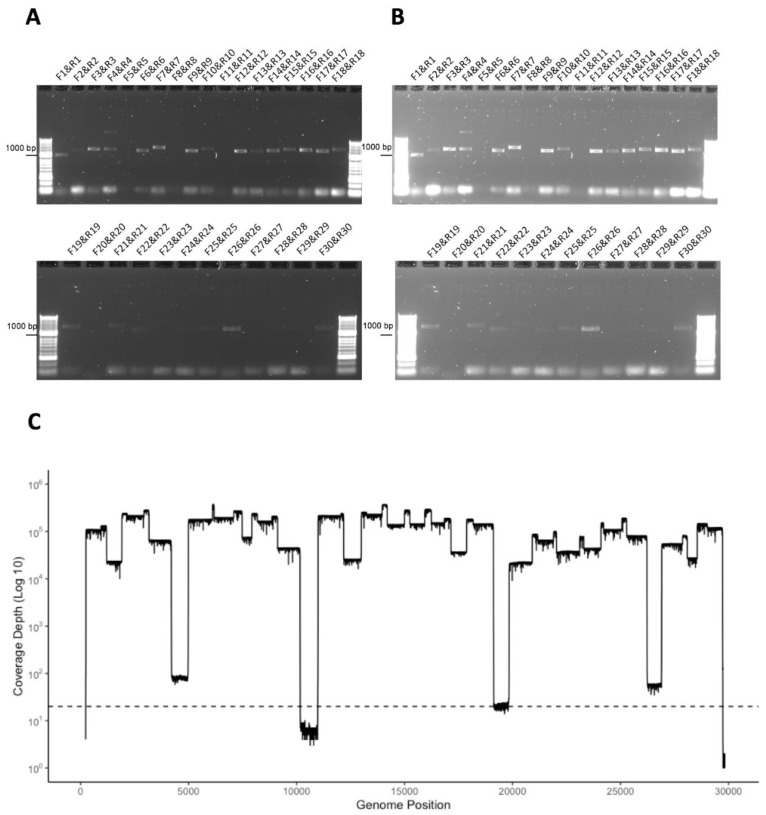
(**A**,**B**) Agarose gel electrophoresis analysis of amplicons generated by RT-PCR from RNA isolated from a nasopharyngeal swab taken from patient REMRQ0002, who had COVID-19, and diagnosed positive for SARS-CoV-2 by a laboratory-based test. Primer pairs are indicated above each amplicon. Note that the image in (**B**) is the same image as (**A**) but the brightness has been enhanced post-image capture in order to more clearly show amplicon products. (**C**) The amplicon products were purified and sequenced on a single flow cell using an Oxford Nanopore MinION. Shown are the number of reads that map (*y*-axis) to each amplicon across the SARS-CoV-2 genome from 5′ to 3′ (*x*-axis).

The second patient (REMRQ0002) had onset of symptoms of COVID-19 on 22 February 2020 and was admitted to the Royal Liverpool University Hospital on 27 February 2020 after a diagnostic nasopharyngeal swab taken on 25 February tested positive for SARS-CoV-2. A repeat swab taken on the day of admission against tested positive for SARS-CoV-2. A further swab was taken for research the same day, and RNA isolated from this swab was used to evaluate the amplicon system. The amplicons were visualised by agarose gel electrophoresis (Figure 3A,B; same gel as shown in 3A but with the brightness enhanced post-image capture). These amplicons were sequenced on an Oxford Nanopore flow cell using MinION and sequence reads were mapped to the SARS-CoV-2 genome (Figure 3C), confirming that the amplicons represented viral sequence. There were some regions of the genome that had lower sequence read depth.

### 3.4. Development of a Multiplex Method to Generate Amplicons

**Figure 4:**
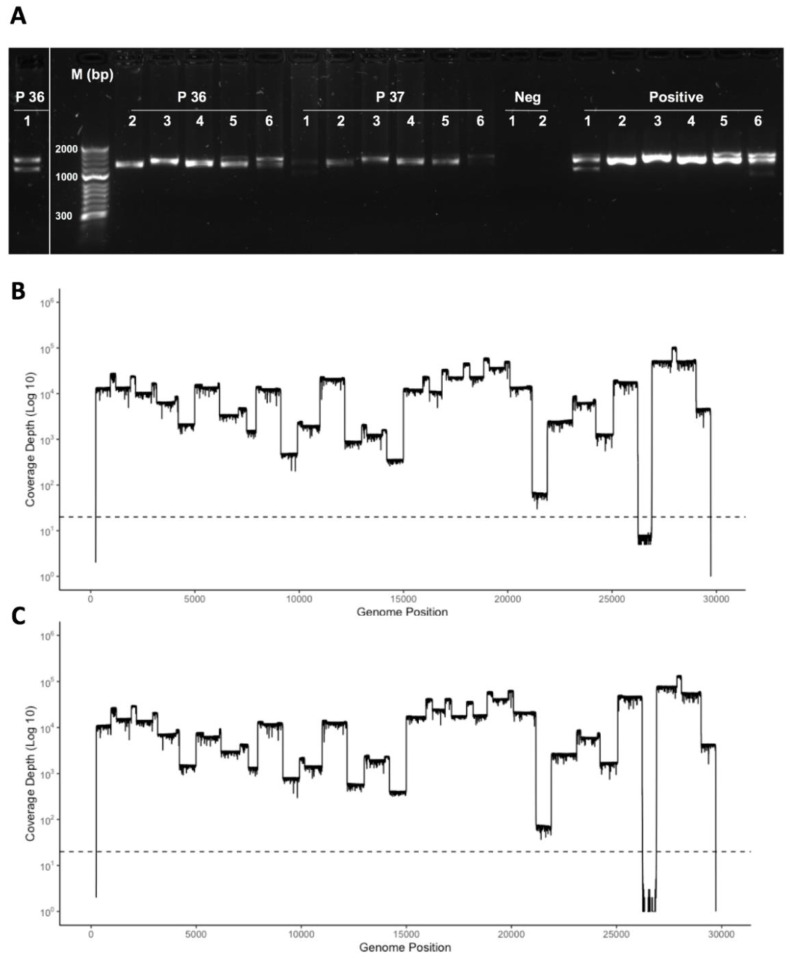
(**A**) Agarose gel electrophoresis analysis of amplicons generated by multiplex RT-PCR from RNA isolated from a nasopharyngeal swab taken from patients who had COVID-19 and diagnosed positive for SARS-CoV-2 by a laboratory-based test. Primer pairs are indicated above each amplicon and exemplar data from two patients (numbers 36 and 37) are shown. Note that amplicons from multiplex pool 1, for patient 36, is shown to the left as these were run on a separate gel. Also shown are negative controls and a positive control using RNA isolated from SARS-CoV-2 infected cells. (**B**,**C**) The amplicon products were purified, barcoded and sequenced on a single flow cell using an Oxford Nanopore MinION. Shown are the number of reads that map (*y*-axis) to each amplicon across the SARS-CoV-2 genome from 5′ to 3′ (*x*-axis).

In order to increase capacity and reduce complexity (the need to conduct individual PCRs for each amplicon primer set), primer sets were pooled into six groups (Table 1). These were arranged so that adjacent regions were not in the same pool and that there was coverage across the genome in each pool. This way, if a reaction failed, there would still be genome coverage in each amplicon group. The ability of this multiplex method to identify SARS-CoV-2 RNA in clinical material was evaluated by isolating RNA from nasopharyngeal swabs taken from patients. Total RNA from SARS-CoV-2 infected cells was also used as a positive control, and negative controls were also included. The six groups of primer sets were used to generate a mixture of amplicons in each pool for each patient and the positive control. These amplicons were visualised by agarose gel electrophoresis (exemplar data shown in Figure 4).

### 3.5. Identification of Deletions in SARS-CoV-2 from Patients with COVID-19

Recombination is a major mechanism of genetic change in coronaviruses, and this will manifest in insertions and deletions being present in the SARS-CoV-2 genome [7]. For coronaviruses in general, this process is well characterised in cell culture and has been demonstrated in vivo for both animal coronaviruses [5] and also in humans for Middle East respiratory syndrome coronavirus (MERS-CoV) [14,15]. To assess whether and where these were occurring during SARS-CoV-2 replication in patients, RNA was extracted from nasopharyngeal swabs taken from 24 patients and amplicons generated using the RSLA multiplex approach and sequenced on Oxford Nanopore flow cells using either MinION and/or GridION. Sequence reads were mapped to the SARS-CoV-2 genome and putative deletions identified (Table 2). Interestingly, several patients had deletions, at the minor variant level, in the SARS-CoV-2 genome scored with high confidence, mainly in *orf3a* and *orf7a*, which are associated with interferon antagonism and would result in potential defective genomes being present.

## 4. Discussion

This work demonstrates that both amplicon-based detection and subsequent sequencing are feasible for identifying the SARS-CoV-2 genome or nucleic acid in samples from patients with COVID-19. The rationale is based on amplifying and sequencing longer fragments than comparative techniques, and our approach was named Rapid Sequencing Long Amplicons (RSLAs) to reflect this and its origins based on identifying MERS-CoV from viral subgenomic messenger RNAs present in clinical samples in Saudi Arabia. The amplicon system was evaluated first on RNA purified from cells infected with SARS-CoV-2 as a positive control and then on RNA purified from nasopharyngeal swabs from patients with COVID-19. We would note that this system best lends itself to identifying and sequencing SARS-CoV-2 from RNA that is of good quality. In general, the Ct value of these diagnostic leftover samples was not known. Different platforms are used in different settings and diagnostic laboratories in the UK, and there is no common uniformity. Interestingly, the RSLA system could identify nucleic acid corresponding to parts of the SARS-CoV-2 genome in a sample from a patient that had tested SARS-CoV-2 negative by an alternative diagnostic nucleic acid-based test performed on a separate sample. Provided that the primer binding sites remain conserved in the pathogen being tested, RT-qPCR is generally more sensitive for diagnostic purposes. In this case, diagnostic reagents for RT-qPCR can be reassessed based upon using sequencing as sentinel for these events. We would note that, based upon our comparison, multiple sites should be targeted within the genome to increase diagnostic sensitivity. Not all regions of the viral genome had equal sequence coverage, and this may be reflective of differential RNA stability in a clinical sample and the gradient of subgenomic messenger RNAs. Nevertheless, there was sufficient coverage to build consensus genomes.

The non-multiplexed RSLA approach has been compared to other viral genome sequencing protocols such as ARTIC and viral bait capture, using the Illumina MiSeq platform, further demonstrating the feasibility and flexibility of this method. Although RSLA generates less depth in comparison to other methods, the breadth of coverage tends to be more even [16]. RNA quality and viral load (Ct values) can influence sequence read depth and obtaining full genome coverage regardless of method. Previous studies on sequencing viral genomes and investigating depth of coverage and accuracy of base calling have shown that MinION sequencing is comparable to short-read sequencing platforms such as Illumina [17,18]. Long-read sequencing data has advantages in detecting insertions and deletions and allows linkage of potentially different sequence variations. These can be missed depending on the type of bioinformatic analysis in short-read data. Much needed information can be gained from sequencing of viral genomes in terms of viral adaptation [9], thus informing molecular epidemiological studies during outbreaks [18]. Current diagnostics may not remain fit for purpose due to recombination in coronaviruses (e.g., [5,19]). Interestingly, analysis of deletions that occurred in the SARS-CoV-2 genome in infected patients from this study identified several that mapped to genes encoding proteins involved in interferon and host antagonism. Deletions with high confidence scores were particularly situated in Orf3a but particularly in Orf7a. These genes are conserved with SARS-CoV [20]. ORF3A in SARS-CoV has been shown to activate the NLRP3 inflammasome [21], induce NF-κB activation and chemokine production [22] and may act as a viroporin. Data from SARS-CoV suggested that ORF7A acts as an RNA silencing suppressor (RSS) and therefore is a suppressor of siRNA activity in mammalian cells [23]. The presence of deletion variants of the genome may also act as defective interfering genomes within patients. Such deletion variants may account for the transient emergence of isolates of SARS-CoV-2 that are associated with the milder disease in patients [24].

While most analysis of genome variation in viruses with RNA genomes is focused on nucleotide substitution, it is recombination in coronaviruses that is one of the big drivers of adaptation and change. A novel genotype of human coronavirus OC43 has been shown to emerge through recombination [25]. Indeed, recombination in coronaviruses can occur between highly divergent coronaviruses of the same strain [26] and even between coronaviruses that infect different species [27], as has been proposed for the origin of these severe coronaviruses in humans. Therefore, we would caution that any surveillance and sequencing pipeline is able to identify recombination and monitor for recombination between SARS-CoV-2 and other coronaviruses—particularly human ones.

## Figures and Tables

**Table 1:** Primer pairs by sequence order.

Primer Name	Sequence 5′–3′	Location		Size (bp)	Multiplex Pool
		Start	End		
SARS-CoV-2_1_F	GTGTGACCGAAAGGTAAGATGG	248	269	956	1
SARS-CoV-2_1_R	TTGCATTCATTTGGTGACGC	1203	1184		
SARS-CoV-2_2_F	GGTGTATACTGCTGCCGTGA	944	963	1213	2
SARS-CoV-2_2_R	GCCAATCAAGGACGGGTTTG	2156	2137		
SARS-CoV-2_3_F	CCGCACTCTTGAAACTGCTC	1912	1931	1254	3
SARS-CoV-2_3_R	GCAGAAGTGGCACCAAATTC	3165	3146		
SARS-CoV-2_4_F	ACACCACTGGGCATTGATTTAG	2936	2957	1264	4
SARS-CoV-2_4_R	TTTCAGTAGTGCCACCAGCC	4199	4180		
SARS-CoV-2_5_F	CTTCATCCAGATTCTGCCAC	4052	4071	1296	5
SARS-CoV-2_5_R	AGCAGGTGGATTAAACTTCAACTC	5347	5324		
SARS-CoV-2_6_F	CAACATTAACCTCCACACGC	4990	5009	1189	6
SARS-CoV-2_6_R	ATCAATAGCCACCACATCACC	6178	6158		
SARS-CoV-2_7_F	AGAAACCTGCTTCAAGAGAGC	6108	6128	1373	1
SARS-CoV-2_7_R	ATTACAACCGTCTACAACATGCAC	7480	7457		
SARS-CoV-2_8_F	GTCACTATTGCAACCTACTGTAC	7091	7113	1093	2
SARS-CoV-2_8_R	CTTGCCGAGCTGCTGAAATA	8183	8164		
SARS-CoV-2_9_F	AATCAGCGTCTGTTTACTACAGTC	7929	7952	1192	3
SARS-CoV-2_9_R	GTGTCAGGGCGTAAACTTTC	9120	9101		
SARS-CoV-2_10_F	TTGTCGTGCCTGGTTTGC	8856	8873	1303	4
SARS-CoV-2_10_R	ACGTCATCAAGCCAAAGACC	10158	10139		
SARS-CoV-2_11_F	AGTGGAGCAATGGATACAAC	9917	9936	1239	5
SARS-CoV-2_11_R	AGCTACAGTGGCAAGAGAAG	11209	11190		
SARS-CoV-2_12_F	AGGGTACACACCACTGGTTG	10995	11014	1185	6
SARS-CoV-2_12_R	CACCATTAGCAACAGCCTGC	12179	12160		
SARS-CoV-2_13_F	GTGAAGAAATGCTGGACAACAG	12057	12078	1180	1
SARS-CoV-2_13_R	GCACCACCAAAGGATTCTTG	13236	13217		
SARS-CoV-2_14_F	TAGTTTAGCTGCCACAGTACG	12997	13017	1200	2
SARS-CoV-2_14_R	AGTTAAAGCCCTGGTCAAGG	14196	14177		
SARS-CoV-2_15_F	ATACGCCAACTTAGGTGAACG	13962	13982	1284	3
SARS-CoV-2_15_R	AACATGTTGTGCCAACCACC	15245	15226		
SARS-CoV-2_16_F	TGAGTTATGAGGATCAAGATGCAC	14996	15019	1243	4
SARS-CoV-2_16_R	GCCTGTAAGACTGTATGCGG	16238	16219		
SARS-CoV-2_17_F	CCCAGATCCATCAAGAATCCTAG	15933	15955	1214	5
SARS-CoV-2_17_R	TGCGAGCAGAAGGGTAGTAG	17146	17127		
SARS-CoV-2_18_F	AAGGTGACTATGGTGATGCTG	16841	16861	1336	6
SARS-CoV-2_18_R	GGTATGCCAGGTATGTCAACAC	18176	18155		
SARS-CoV-2_19_F	ACTCAAACCACTGAAACAGCTC	17875	17896	1239	1
SARS-CoV-2_19_R	GTCACTACAAGGCTGTGCATC	19113	19093		
SARS-CoV-2_20_F	AGCTAGTTGTGATGCAATCATGAC	18846	18869	1235	2
SARS-CoV-2_20_R	CTTGTTTGGGACCTACAGATGG	20098	20077		
SARS-CoV-2_21_F	TTTGGGTGTGGACATTGCTG	19842	19861	1323	3
SARS-CoV-2_21_R	ATAGCCACGGAACCTCCAAG	21164	21145		
SARS-CoV-2_22_F	TAAGACAGTGGTTGCCTACG	20912	20931	1125	4
SARS-CoV-2_22_R	TCTGAACTCACTTTCCATCCAAC	22036	22014		
SARS-CoV-2_23_F	TTCGAAGACCCAGTCCCTAC	21895	21914	1405	5
SARS-CoV-2_23_R	TGGATCACGGACAGCATCAG	23299	23280		
SARS-CoV-2_24_F	TTGAACTTCTACATGCACCAGC	23106	23127	1111	6
SARS-CoV-2_24_R	CCAGAAGTGATTGTACCCGC	24216	24197		
SARS-CoV-2_25_F	TTGCTGCTAGAGACCTCATTTG	24093	24114	1190	1
SARS-CoV-2_25_R	GCAACTGGTCATACAGCAAAG	25282	25262		
SARS-CoV-2_26_F	GGTGACATCTCTGGCATTAATGC	25061	25083	1163	2
SARS-CoV-2_26_R	TGCTTACAAAGGCACGCTAG	26223	26204		
SARS-CoV-2_27_F	ACCAGCTGTACTCAACTCAATTG	26027	26049	1137	3
SARS-CoV-2_27_R	CTGCTACTGGAATGGTCTGTG	27163	27143		
SARS-CoV-2_28_F	TGACCAGACCGCTTCTAGAAAG	26908	26929	1180	4
SARS-CoV-2_28_R	GCCTCATCCACGCACAATTC	28087	28068		
SARS-CoV-2_29_F	TGTCACGCCTAAACGAACATG	27876	27896	1147	5
SARS-CoV-2_29_R	GATTTCTTAGTGACAGTTTGGCC	29022	29000		
SARS-CoV-2_30_F	CGAATTCGTGGTGGTGACG	28550	28568	1173	6
SARS-CoV-2_30_R	GGTGGCTCTTTCAAGTCCTC	29722	29703		

**Table 2:** Analysis of deletions in the SARS-CoV-2 genome in patients with COVID-19. The columns from left to right are as follows: sample barcode, deletion start position (bp), deletion end position (bp), number of reads supporting this deletion, quality score (similar to number of reads supporting, but also takes into account read mapping quality scores with a score greater than 10 having higher confidence), standard deviation (SD) of deletion span (bp) from supporting reads, SD of deletion position (bp) from supporting reads. If the deletion interrupts a gene, these are the coordinates of the gene, the gene name, and the bp overlap with the deletion. In cases where the deletion overlaps >1 gene, the information of the second gene is provided.

Deletion Information							Affected Gene Information							
Patient Number	Start (bp)	End (bp)	Supporting Reads	Quality Score	SD Span	SD Pos	Gene Start	Gene End	Gene Name	Overlap (bp)	Gene2 Start	Gene2 End	Gene2 Name	Overlap2 (bp)
22	19325	19380	9	10	4.42	58.42	266	21555	*Orf1ab*	55	−	−	−	−
22	20294	20429	8	10	0	0	266	21555	*Orf1ab*	135	−	−	−	−
22	25417	25796	10	12	0.79	0.86	25393	26220	*Orf3a*	379	−	−	−	−
22	27578	27624	16	19	2.49	2.45	27394	27759	*Orf7a*	46	−	−	−	−
22	28756	28884	7	8	2.27	3.4	28274	29533	*N*	128	−	−	−	−
23	2143	2198	5	5	4.36	73.69	266	21555	*Orf1ab*	55	−	−	−	−
23	2375	2421	8	8	5.54	82.85	266	21555	*Orf1ab*	46	−	−	−	−
23	2589	2642	6	6	3.2	41.78	266	21555	*Orf1ab*	53	−	−	−	−
23	2654	2741	5	5	8.73	51.88	266	21555	*Orf1ab*	87	−	−	−	−
23	2859	2904	9	10	2.42	88.48	266	21555	*Orf1ab*	45	−	−	−	−
25	20274	20383	7	8	6.2	7.5	266	21555	*Orf1ab*	109	−	−	−	−
25	20279	20340	10	12	3.83	2.57	266	21555	*Orf1ab*	61	−	−	−	−
25	27594	27640	10	11	2.37	31.77	27394	27759	*Orf7a*	46	−	−	−	−
26	27386	27699	91	99	0.86	1.66	27394	27759	*Orf7a*	305	27202	27387	*ORF6*	1
26	20274	20338	9	11	1.5	1.02	266	21555	*Orf1ab*	64	−	−	−	−
27	27020	27073	11	13	0.6	0.3	26523	27191	*M*	53	−	−	−	−
27	27522	27761	24	29	1.09	2.04	27394	27759	*Orf7a*	237	−	−	−	−
27	27689	27763	23	27	3.58	29.9	27394	27759	*Orf7a*	70				
28	2025	2088	5	5	7.16	80.94	266	21555	*Orf1ab*	63				
28	25508	25568	6	6	8.31	61.45	25393	26220	*Orf3a*	60				
28	27884	27934	6	6	7.19	27.43	27894	28259	*Orf8*	40				
29	27546	27650	7	8	14.61	64.8	27394	27759	*Orf7a*	104				
29	28454	28731	6	7	26.89	58.79	28274	29533	*N*	277				
30	25398	25776	12	14	1.56	69.87	25393	26220	*Orf3a*	378				
30	27555	27625	31	38	1.94	5.71	27394	27759	*Orf7a*	70				
30	8676	8723	11	12	1.79	38.61	266	21555	*Orf1ab*	47				
31	28444	28775	62	77	1.29	1.31	28274	29533	*N*	331				
32	25480	25551	97	99	1.62	2.04	25393	26220	*Orf3a*	71				
36	20274	20339	11	13	1.43	0.87	266	21555	*Orf1ab*	65				
37	25429	25641	11	13	17	7.89	25393	26220	*Orf3a*	212				
37	25432	25808	10	12	5.24	44.62	25393	26220	*Orf3a*	376				
37	28444	28776	8	9	0.71	0.65	28274	29533	*N*	332				
43	27426	27559	5	5	19.77	50.25	27394	27759	*Orf7a*	133				
43	27690	27732	6	6	2.25	49.85	27394	27759	*Orf7a*	42				
43	28011	28062	13	14	5.36	77.82	27894	28259	*Orf8*	51				
43	28196	28238	5	5	2	96.73	27894	28259	*Orf8*	42				
43	28481	28536	5	5	3	64.73	28274	29533	*N*	55				
	28601	28718	5	5	4.82	43.76	28274	29533	*N*	117				
